# The Gut Microbiome of the Goitered Gazelle Enables Plasticity by Responding to Environmental Factors in the Qaidam Basin

**DOI:** 10.3390/biology15020118

**Published:** 2026-01-07

**Authors:** Qing Zhao, Bin Li, Juan Ma, Jiaxin Wei, Wen Qin

**Affiliations:** 1 State Key Laboratory of Plateau Ecology and Agriculture, Qinghai University, Xining 810016, China; zhaoqing1063@163.com (Q.Z.); 15682920216@163.com (J.M.); weijiaxinyouxiang@163.com (J.W.); 2 School of Ecological and Environmental Engineering, Qinghai University, Xining 810016, China; 3 Key Laboratory of Adaptation and Evolution of Plateau Biota, Northwest Institute of Plateau Biology, Chinese Academy of Sciences, Xining 810008, China; libin@nwipb.cas.cn

**Keywords:** *Gazella subgutturosa*, gut microbiome, the Qaidam Basin, environmental factors

## Abstract

The Qaidam Basin on the Qinghai–Tibet Plateau is an extreme arid environment, posing severe survival challenges. The goitered gazelle (*Gazella subgutturosa*) is a keystone species in this fragile ecosystem, yet the ecological role of its gut microbiota and its associations with environmental drivers remain poorly understood. We collected fecal samples from gazelles across seven regions of the basin. Metagenomic sequencing was employed to characterize the gut microbiome. Statistical analyses (Mantel tests, multiple regression on matrices, co-occurrence networks) were used to link microbial composition and function with key environmental factors. The gut microbiota was dominated by fiber-degrading phyla (*Firmicutes*, *Bacteroidota*) and enriched in metabolic pathways, aligning with a high-fiber diet. Regarding environmental drivers of gut microbial composition variations, isothermality and soil organic carbon were significant predictors, likely via vegetation and environmental inoculation. Regarding environmental drivers of gut microbial function variations, winter solar radiation was uniquely associated with metabolic function without altering microbial composition, suggesting a functional plasticity—the capacity to shift metabolic profiles independently of taxonomic turnover—in response to environmental variation. The gut microbiota of the goitered gazelle exhibits a stable core composition alongside environmentally responsive functional modules. This suggests the microbiome may serve as a significant mediator of host resilience, highlighting adaptation as a dynamic interplay between host, microbiome, and environment. These insights are crucial for microbiome-assisted conservation.

## Simple Summary

This study investigated the environment-associated variation in the gut microbiota in the goitered gazelle (*Gazella subgutturosa*) inhabiting the extreme arid environment of the Qaidam Basin on the Qinghai–Tibet Plateau, and identified the key environmental drivers involved. Using metagenomic sequencing and multivariate statistical analysis, we found that the gazelle’s gut microbiome was dominated by the fiber-degrading phyla *Firmicutes* and *Bacteroidota* and enriched in functions such as carbohydrate metabolism, aligning with its high-fiber diet. Environmental analysis revealed that isothermality and soil organic carbon content were statistically associated with microbial community structure, likely operating indirectly by modulating vegetation quality and environmental microbial inoculation. Notably, winter solar radiation specifically influenced microbial metabolic function without altering community composition, suggesting potential functional plasticity—defined as the capacity to modulate metabolic outputs—in response to environmental variation. In conclusion, the gut microbiota of the goitered gazelle appears to maintain core functional stability while exhibiting the flexibility to adjust specific functional modules to cope with varying environmental conditions. This expands our perspective on the potential role of the microbiome in the dynamic interplay between host, microbiome, and environment, providing valuable insights for assessing ecological resilience and developing microbiome-informed conservation strategies for endangered species.

## 1. Introduction

The Qinghai–Tibet Plateau, renowned as the “Roof of the World”, harbors a unique and fragile ecosystem and is recognized as a biodiversity hotspot [1]. Its harsh conditions—characterized by cold, low oxygen, and strong UV radiation—pose significant challenges yet simultaneously drive unique adaptations [2]. A prime example of such an extreme environment is the Qaidam Basin, located in the northeastern part of the Qinghai–Tibet Plateau. As a cold, arid inland basin with an average altitude of 2600–3000 m, it receives less than 100 mm of annual precipitation while experiencing evaporation exceeding 2000 mm. Consequently, the basin is subject to intense solar radiation, severe aridity, and dramatic temperature fluctuations [3], resulting in sparse vegetation, low species diversity, and limited primary productivity, and thus forming a highly fragile ecosystem [4]. Therefore, the Qaidam Basin represents a critical area for studying the ecological resilience and environment-associated variation in wildlife under extreme environmental stress.

The goitered gazelle (*Gazella subgutturosa*) is the only wild ungulate species currently found in the core area of the Qaidam Basin. It primarily feeds on xerophytic plants such as *Haloxylon ammodendron*, *Ceratoides latens*, and *Kalidium foliatum*, and can survive on metabolic water derived from food in the absence of surface water sources [5]. As the only medium- to large-sized primary consumer in the region, the goitered gazelle plays a key role in maintaining material cycling and energy flow in the Qaidam Basin. It is therefore an important indicator species for monitoring the structure and dynamics of this fragile desert environment.

In recent years, the gut microbiota of animals, often referred to as the “second genome,” has drawn increasing attention for its crucial role in host adaptation to environmental changes. These microbial communities are integral to host physiology, participating extensively in nutrient metabolism, immune regulation, and oxidative-stress responses, largely through their diverse metabolic products [6]. For wild ungulates inhabiting nutrient-poor environments, key gut microbial functions—such as the degradation of low-quality plant cellulose, synthesis of short-chain fatty acids (SCFAs), and efficient nitrogen cycling—are considered vital survival strategies [7]. Supporting this, metagenomic studies on the goitered gazelle specifically indicate that its gut microbial community is enriched with functional genes related to high salt tolerance, lignocellulose degradation, and enhanced energy recovery [8]. This suggests the presence of a gut microbial community functionally suited to extreme environments of the Qaidam Basin.

Moreover, environmental factors are crucial external drivers shaping the composition and function of the gut microbiota. For example, altitude, temperature, precipitation, soil pH, and vegetation structure can influence food availability and host metabolic states, thereby indirectly regulating the structure and stability of gut microbial communities [9,10]. In cold and arid environments, wild herbivores often exhibit reduced gut microbial diversity alongside increasingly specialized metabolic functions, which collectively enhance their ability to cope with environmental stresses [11]. However, research on the regional variation and functional response mechanisms of ungulate gut microbiomes in typical arid ecosystems remains limited, particularly in the Qaidam Basin. Importantly, interpreting gut microbiome patterns in such wild populations requires careful consideration of several methodological and ecological constraints. These include distinguishing between signals from different gut compartments (e.g., rumen versus feces), accounting for seasonal shifts in diet and their confounding effects with abiotic factors, and the challenge of isolating purely environmental influences from host-driven dietary adaptations.

Furthermore, from a conservation perspective, the goitered gazelle (a Class II protected species in China) faces mounting threats from climate change, habitat fragmentation, and human activity on the Qinghai–Tibet Plateau [12,13]. Therefore, moving beyond merely cataloging microbial composition to elucidate the environment-microbiome-host adaptation axis is not only a key ecological question but also a conservation imperative. Clarifying this can help us better predict the impacts of climate change on endangered populations and inform microbiome-based interventions to enhance individual resilience and support ecosystem restoration.

To address this gap, this study investigates the gut microbiota of the goitered gazelle across different sampling sites in the Qaidam Basin. We hypothesize that: (1) environmental factors differentially influence the gut microbiota of goitered gazelle, with one or a few key drivers playing a dominant role; and (2) the gut microbiota can maintain host homeostasis through compositional and functional plasticity, thereby enabling its survival in the extreme environment of the Qaidam Basin. By integrating environmental data with host-associated metagenomics, this work seeks to provide a holistic understanding of the survival strategy of this keystone species in one of the world’s most challenging ecosystems. The findings are expected to deepen our knowledge of wildlife–microbiome–environment interactions and provide essential scientific support for the conservation of endangered species, the maintenance of ecosystem stability, and regional sustainable development on the Qinghai–Tibet Plateau. 

## 2. Materials and Methods

### 2.1. Fecal Sample Collection

**Figure 1:**
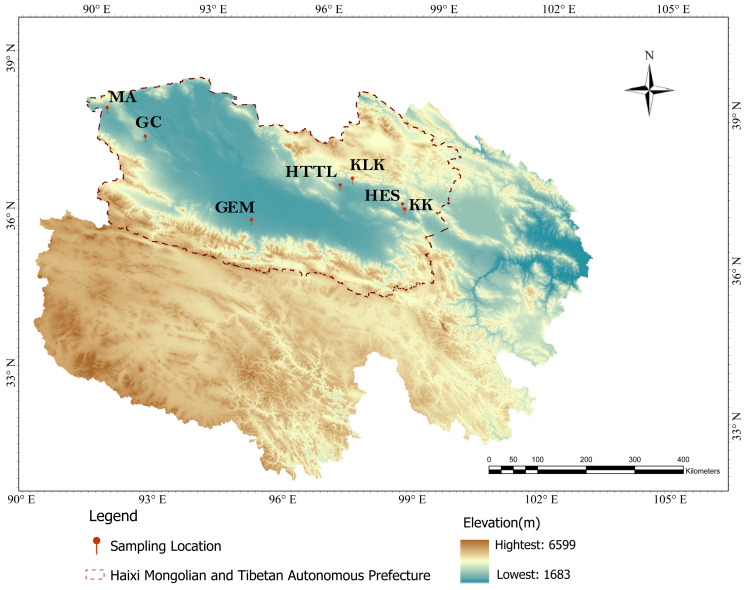
Sampling regions of goitered gazelles in Qinghai Province, China.

In March 2023, forty-four fresh fecal samples from wild goitered gazelles were collected at seven sites in the Qaidam Basin of Qinghai Province: KK (KeKe, *n* = 5), KLK (KeLuKe, *n* = 5), HES (HaErSi, *n* = 8), HTTL (HuaiTuTeLa, *n* = 5), GC (GangCi, *n* = 8), GEM (GeErMu, *n* = 5), and MA (MangAi, *n* = 8), respectively (Figure 1). All fecal samples were collected from the spots where goitered gazelle populations were observed, with each site’s samples completed within a single day. We selected relatively intact fecal pellets for collection. To minimize soil contamination, disposable polyethylene (PE) gloves were worn and replaced between each sample. Each collected sample was first placed in a self-sealing bag, then wrapped in aluminum foil, immediately stored in a portable liquid nitrogen tank, and subsequently transported to the laboratory.

### 2.2. Metagenomic Sequencing and Annotation

Total genomic DNA was extracted from each sample using the E.Z.N.A. soil DNA Kit (Omega Bio-tek, Norcross, GA, USA) following the manufacturer’s instructions. DNA concentration and purity were assessed using NanoDrop 2000 (ThermoFisher, Waltham, MA, USA), and DNA quality was verified as 1% agarose gel. After extraction, the DNA was fragmented using Covaris M220 (Gene Company, Hong Kong, China) and fragments of approximately 400 bp were selected for constructing paired-end read (PE) libraries. Paired-end libraries were prepared using the NEXTFLEX Rapid DNA-Seq (Bioo Scientific, Austin, TX, USA). Adapters containing the full set of sequencing primer hybridization sites were ligated to the blunt ends of the fragments. Metagenomic sequencing was performed on an Illumina NovaSeq platform (Illumina, San Diego, CA, USA) at Majorbio Bio-Pharm Technology Co., Ltd. (Shanghai, China) and Shanghai Personal Biotechnology Co., Ltd. (Shanghai, China).

FASTP (v 0.20.0) was used to perform quality control and host filtering on the raw reads obtained from metagenomic sequencing data [14]. Subsequently, BWA (v 0.7.18) was used to remove reads with high similarity to the host genome to avoid contamination [15]. A total of 195,714,780 optimized reads were obtained. The optimized sequences were assembled using MEGAHIT (v 1.1.2) software, and contigs with lengths ≥ 300 bp were selected as the final assembly [16]. Contigs with nucleotide lengths ≥ 100 bp from the assembly results were subjected to ORF prediction using prodigal (v 2.6.3), followed by translation into amino acid sequences [17]. The predicted gene sequences were clustered using CD-HIT (v4.8.1) to construct a non-redundant gene set, with clustering criteria set at a sequence similarity identity of at least 0.9 and a coverage of at least 0.9 [18]. High-quality reads were then aligned to the non-redundant gene catalog to calculate gene abundance with 95% identity using SOAPaligner (v 2.21) [19]. Representative sequences from the non-redundant gene catalog were aligned to the NCBI non-redundant protein (NR) database with an e-value cutoff of 1 × 10^−5^ using Diamond (v 2.0.13) [20] (https://ftp.ncbi.nlm.nih.gov/blast/db/FASTA/, accessed on 10 January 2025) for taxonomic annotation. KEGG annotation was performed using the same Diamond tool against the KEGG database [21] (http://www.kegg.jp, accessed on 10 January 2025) with an e-value cutoff of 1 × 10^−5^. Metagenomic sequencing and annotation were carried out by Majorbio Bio-Pharm Technology Co., Ltd. (Shanghai, China). Through these annotations, the species, functional, and resistance gene abundances at various levels were obtained, and statistical analyses were performed based on the abundance data.

### 2.3. Collection and Processing of Environmental Data

A total of 161 environmental factors were collected for this study. All factors were sourced from public databases and are widely used in species distribution modeling [22,23]. The dataset included: (1) 19 bioclimatic factors (bio_01–bio_19) from WorldClim (http://www.worldclim.org, accessed on 21 June 2024); (2) monthly data for precipitation, solar radiation, temperature (mean, minimum, and maximum), and elevation [24]; (3) layers for China’s vegetation types, population density, the human footprint index [25], the normalized difference vegetation index (NDVI), and land-use types, obtained from the China Resource and Environment Data Center (http://www.resdc.cn/, accessed on 29 July 2024), the National Earth System Science Data Sharing Platform (http://www.gscloud.cn/, accessed on 30 July 2024), and the National Space Science Data Center (http://www.dsac.cn/, accessed on 31 July 2024), respectively; (4) 14 spatial heterogeneity metric layers quantifying habitat heterogeneity from the Global Habitat Heterogeneity Database (https://www.earthenv.org/, accessed on 11 August 2024) [26]; and (5) 61 soil property layers (e.g., bulk density of the fine earth fraction) from the Global Soil Information System SoilGrids (https://soilgrids.org/, accessed on 15 August 2024) at a 1 km^2^ resolution [27].

In ArcGIS Pro version 3.4.3 (Redlands, CA, USA), the bio_01 raster layer was clipped using the vector boundary of Qinghai Province. Subsequently, in R (v 4.5.1), the remaining layers were resampled with the resample function from the raster package [28], using the clipped bio_01 layer serving as the template. All layers were resampled to match the spatial extent and resolution of bio_01, using bilinear interpolation as the resampling method. Finally, environmental values for each of the 161 factors were extracted at the 44 validated goitered gazelle sampling coordinates using the extract function from the same raster package of the goitered gazelles [28].

### 2.4. Statistical Data Analysis

All statistical analyses were conducted using R (v 4.3.2). The Kruskal–Wallis test was applied to assess differences in microbial diversity, including Shannon index and Chao index between groups at a 95% confidence level. False discovery rate (FDR) correction was performed for multiple comparisons [29], and Dunn’s test (α = 0.05) was used for post hoc analysis. Next, we used the mothur, boot, and stats packages [30,31,32] to calculate the diversity of microbiota. For beta-diversity analysis, community dissimilarity was quantified based on the Bray–Curtis distance matrix using the vegan package [33], followed by ANOSIM analysis and permutational multivariate analysis of variance (PERMANOVA; 999 permutations) to assess intergroup differences. Analysis of multivariate dispersion (betadisper; 999 permutations) was used to check the homogeneity of group dispersions. Non-metric Multidimensional Scaling (NMDS) analyses were performed for visualizing community structure differences [34]. We used LEfSe software (http://galaxy.biobakery.org/, accessed on 16 August 2025) for differential analysis of gut microbiota [35], and Linear Discriminant Analysis (LDA) to estimate the impact of each differential species or function on the intergroup differences.

Using Mantel test [36], we evaluated whether the variation in gut microbial composition and functional categories of goitered gazelles was significantly correlated with the environmental factors or predictor factors of their habitats. Environmental variables were chosen for their ecological relevance, including climate, soil, vegetation, and land use factors. Dissimilarity matrices for each environmental factor or predictor variable were calculated using the vegan package [33] in R (with Bray–Curtis distances applied to habitat suitability index, ldcv, and veg, and Euclidean distances applied to the remaining factors). These distance matrices were then used for the Mantel test, and correlations with FDR-adjusted *p* < 0.05 were considered significant. Environmental factors that showed significant correlations (method = “pearson”, *p* < 0.05) in the Mantel tests were selected as key predictors for subsequent modeling [17]. To reduce the impact of multicollinearity on subsequent predictive modeling, environmental variables significantly associated with the gut microbiota composition and function of the goitered gazelle were subjected to hierarchical clustering based on Spearman correlations. Variables with a high degree of correlation (|*ρ*| ≥ 0.7) were removed. During variable selection, we followed the principle of prioritizing factors that could maximally explain the variation in other correlated variables. Prior to analysis, these predictor factors were standardized using the stdize function from the MuMIn package (v 1.48.11) [37]. Subsequently, the MRM predictive model was constructed using the ecodist package (v 2.1.3) [38] with 999 permutations (mRank = TRUE; nperm = 999). The trans_network function in the microeco package (v 1.15.0) [39] was used to construct networks based on the genus-level gut microbial composition and the KEGG level 2 functional abundance in goitered gazelles. During network construction, the SparCC algorithm in the SpiecEasi package (v 1.99.0) [40] was applied, retaining only edges with significant correlations (*p* < 0.01). The optimal threshold for correlation coefficients was determined using Random Matrix Theory (RMT). The co-occurrence networks constructed above were visualized using Gephi (v 0.9.2), and the PageRank algorithm [41] (probability = 0.85; tolerance = 0.001; edge weights considered during calculation) was applied to identify the core genera and functional categories within the networks.

## 3. Results

### 3.1. Characteristics of the Metagenomic Data

**Figure 2:**
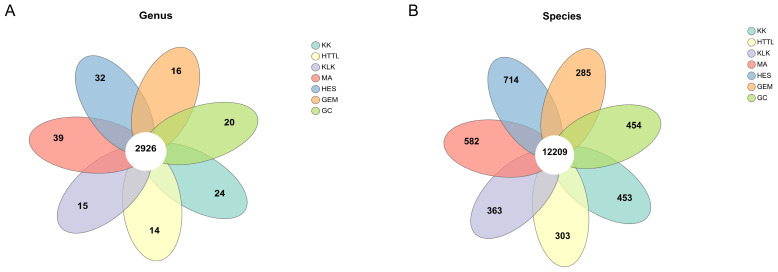
Venn diagram of the gut microbiota of seven groups, with taxonomic characterization at (**A**) the genus level and (**B**) the species level.

Sequencing of 44 samples yielded a total of 797.720 Gb of raw data, with an average of 18.130 Gb per sample. After quality control, 797.539 Gb of clean data were obtained, averaging 18.126 Gb per sample. The clean data accounted for 99.98% of the raw data, indicating that it met the quality requirements for subsequent analysis. Taxonomic classification identified spanning 158 phyla, 267 classes, 487 orders, 1008 families, 4212 genera and 27,451 species. Based on the Venn diagram analysis, at the genus level, 3500 genera were shared across the gut microbiota of goitered gazelles from all seven regions, with 140 genera unique to the HES group and 81 genera unique to the MA group (Figure 2A). At the species level, a total of 13,532 species were shared among the seven regions (Figure 2B), with the HES group having the highest number of unique species (*n* = 1020), followed by the MA group (*n* = 738) and the KK group (*n* = 635).

### 3.2. Gut Microbiome Composition

**Figure 3:**
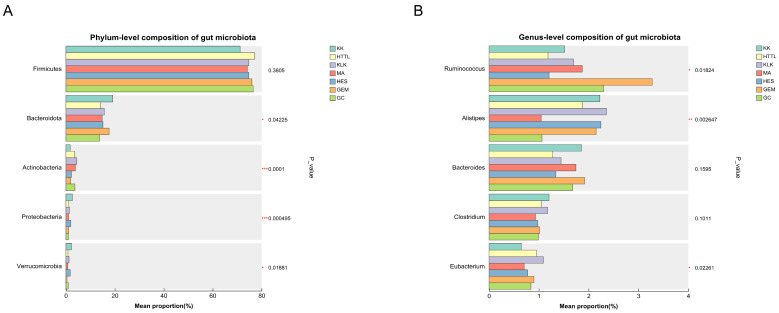
The top five (**A**) phyla and (**B**) genera in relative abundance of gut microbiota among seven regions based on the Kruskal–Wallis test (* represents *p* < 0.05; ** represents *p* < 0.01; *** represents *p* < 0.001).

Based on sequencing results, the relative abundances of gut microbiota were calculated at the phylum and genus levels for the seven groups (Figure 3). In the gut microbiome of goitered gazelles, the five most abundant phyla were *Firmicutes* (74.98%  ±  3.77%), *Bacteroidota* (15.49%  ±  3.22%), *Actinobacteriota* (3.10%  ±  1.36%), *Proteobacteria* (1.49%  ±  0.67%), and *Verrucomicrobiota* (1.19%  ±  1.00%). Among these, *Firmicutes* and *Bacteroidota* were dominant, with relative abundances exceeding 65.29% and 10.45% across all groups, respectively. At the genus level, the five most abundant genera were *Ruminococcus* (1.84%  ±  1.01%), *Alistipes* (1.77%  ±  0.85%), *Bacteroides* (1.60%  ±  0.53%), *Clostridium* (1.03%  ±  0.19%), and *Eubacterium* (0.83%  ±  0.23%). Among them, *Ruminococcus* (*p* < 0.05), *Alistipes* (*p* < 0.01), and *Eubacterium* (*p* < 0.05) showed significant differences among the seven regions.

### 3.3. Gut Microbial Analysis at α- and β-Diversity Levels

**Figure 4:**
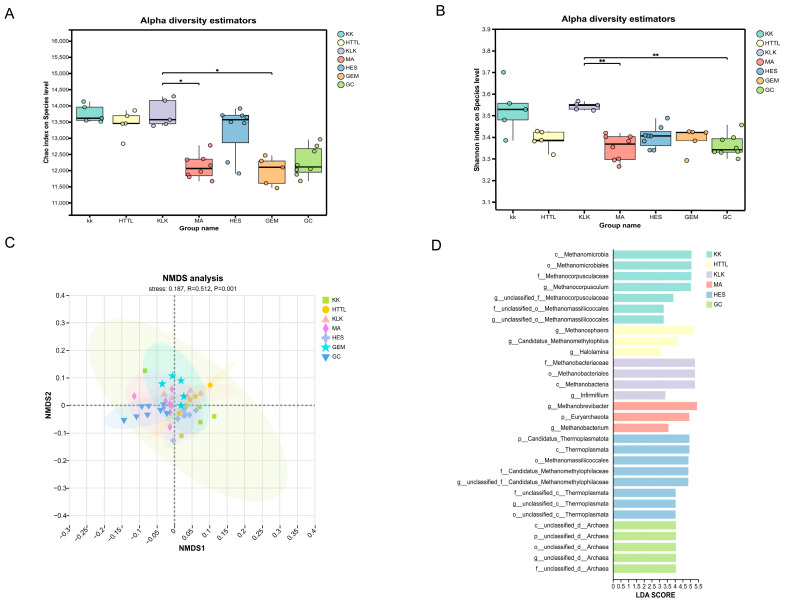
The α-diversity of the (**A**) Chao1 index and (**B**) Shannon index in gut microbiota at the species level among seven groups based on the Kruskal–Wallis test. (**C**) Non-metric multidimensional scaling (NMDS) plot showing gut microbial community structure. (**D**) Histogram of linear discriminant analysis (LDA) scores identifying group-specific microbial biomarkers (LDA score > 3.0). (*: *p* < 0.05, **: *p* < 0.01.). Groups: KK (*n* = 5), HTTL (*n* = 5), KLK (*n* = 5), MA (*n* = 8), HES (*n* = 8), GEM (*n* = 5), GC (*n* = 8).

Comparative analyses of gut microbial diversity at the species level revealed differences in community composition and structure among the seven groups of goitered gazelles. At α-diversity level, it showed that the KLK group exhibited the highest Shannon and Chao1 indices at the species level, whereas the MA group had the lowest gut microbial α-diversity (Figure 4A,B). These differences were statistically significant (*p* < 0.05), indicating that the MA group harbored markedly lower gut microbial diversity compared to the KLK group.

Regarding β-diversity, in terms of β-diversity, the permutation test for homogeneity of multivariate dispersions revealed no statistically significant differences in dispersion among groups (F value = 1.593, *p* = 0.181), satisfying the prerequisite for PERMANOVA. PERMANOVA analysis indicated significant differences in gut microbial composition among the seven groups (R^2^ = 0.429, *p* = 0.001), which was further supported by ANOSIM results (R = 0.512, *p* = 0.001). NMDS ordination showed a stress value of 0.187 (*p* = 0.001), confirming that gut microbial community structures differed significantly among the seven groups, with greater variation between groups than within groups (Figure 4C). To identify specific taxa driving the inter-group differences, we performed Linear Discriminant Analysis Effect Size (LEfSe) analysis. This revealed 30 bacterial biomarkers with LDA scores > 3.0 and *p* < 0.05. Notably, the HES group harbored the highest number of these discriminant biomarkers, while the MA group had the fewest (Figure 4D).

### 3.4. Gut Microbial Functional Analysis

**Figure 5:**
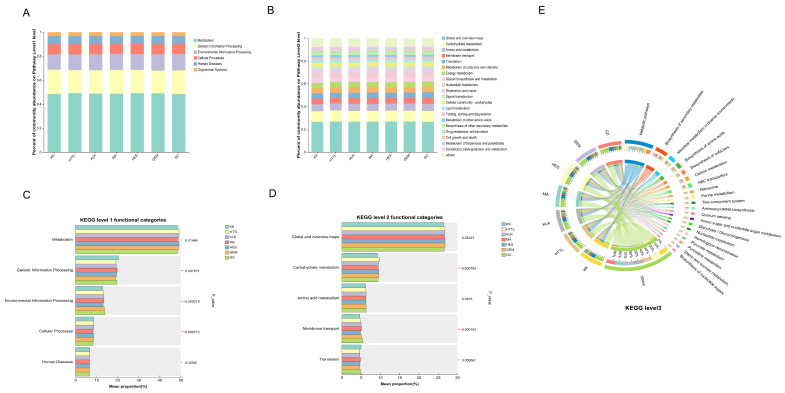
Gut microbial functional composition across seven sampling regions. (**A**,**B**) Relative abundance of functional categories at KEGG level 1 (**A**) and level 2 (**B**). (**C**,**D**) Comparison of the five most abundant categories at each corresponding level ((**C**): level 1; (**D**): level 2) among regions (Kruskal–Wallis test). (*: *p* < 0.05, **: *p* < 0.01, ***: *p* < 0.001.) (**E**) Circos plot showing the relative abundance of major KEGG level 3 pathways. Groups: KK (*n* = 5), HTTL (*n* = 5), KLK (*n* = 5), MA (*n* = 8), HES (*n* = 8), GEM (*n* = 5), GC (*n* = 8).

To investigate the functional differences in gut microbiota among goitered gazelles from different regions, a total of 195,714,780 UniGenes obtained from metagenomic sequencing were annotated using the KEGG database. Among these, 9,325,868 (4.765%) UniGenes were successfully annotated and classified into 10,636 KEGG ortholog groups, while 579,953 UniGenes (6.22% of annotated genes; 0.30% of total genes) were mapped to 458 KEGG pathways. KEGG functional annotation revealed that the gut microbiota of goitered gazelles was primarily enriched in seven major categories at the KEGG level 1 (Figure 5A), including Metabolism (48.77% ± 0.7%), Genetic Information Processing (19.57% ± 0.65%), Environmental Information Processing (13.38% ± 0.47%), Cellular Processes (8.45% ± 0.18%), Human Diseases (6.65% ± 0.1%), and Organismal Systems (3.18% ± 0.05%) (Figure 5C).

At the KEGG level 2 (Figure 5B), the most abundant functions across the gut microbiota of wild goitered gazelles were Global and Overview Maps (26.68% ± 0.25%), Carbohydrate Metabolism (9.48% ± 0.2%), Amino Acid Metabolism (6.26% ± 0.012%), Membrane Transport (5.04% ± 0.29%), and Translation (4.78% ± 0.23%). Notably, among the top five dominant second-level pathways, Carbohydrate Metabolism (*p* < 0.01), Membrane Transport (*p* < 0.01), and Translation (*p* < 0.01) showed significant differences among regions, whereas Global and Overview Maps and Amino Acid Metabolism showed no significant differences across the seven regions (Figure 5D).

At the KEGG level 3 (Figure 5E), the functional profiling of goitered gazelle gut microbiota indicated that the most abundant pathways, in descending order, were Metabolic Pathways (17.76% ± 0.13%), Biosynthesis of Secondary Metabolites (8.38% ± 0.07%), Microbial Metabolism in Diverse Environments (4.73% ± 0.10%), Biosynthesis of Amino Acids (3.94% ± 0.07%), and Biosynthesis of Cofactors (2.92% ± 0.08%).

### 3.5. Environmental Drivers of Gut Microbial Composition Variations

**Figure 6:**
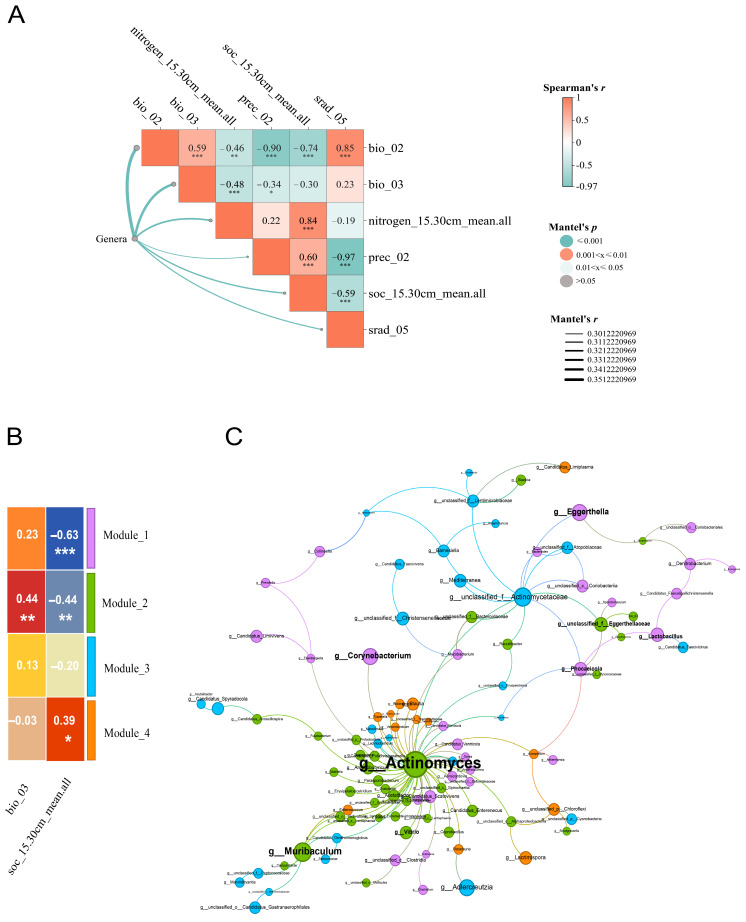
(**A**) Environmental factors significantly correlated with variation in the gut microbial composition of goitered gazelles. (**B**) Spearman correlation heatmap between the eigengenes of the identified network modules and bio_03, soc_15.30cm_mean.all, where * *p* < 0.05, ** *p* < 0.01, *** *p* < 0.001. (**C**) Co-occurrence network of genus-level gut microbial composition in goitered gazelles. Node colors indicate the module to which each node belongs, and node size and label font size represent the node’s PageRank value, reflecting its overall importance within the network.

The associations between multiple environmental factors and the genus-level gut microbial composition (based on Bray–Curtis distances) in goitered gazelles were statistically evaluated using Mantel tests (Figure 6A). Six factors showed significant correlations (Mantel test, *p* < 0.05), including soil nitrogen (nitrogen_15.30cm_mean.all), soil organic carbon (soc_15.30cm_mean.all), temperature-related factors (bio_02, bio_03), precipitation (prec_02), and solar radiation (srad_05). To reduce multicollinearity in the predictive model, we filtered the environmental factors by grouping them based on Spearman correlation and retaining the most explanatory factors. Consequently, three factors—isothermality (bio_03), soil organic carbon (soc_15.30cm_mean.all), and soil nitrogen (nitrogen_15.30cm_mean.all)—were selected for subsequent predictive modeling.

To predict the variation in gut microbial community structure across goitered gazelle populations, we constructed a multiple regression on matrices (MRM) model, applying a stepwise variable selection strategy. Results indicated that model 1 and model 2 explaining 19.1% and 19.0% of the variation (Table 1), respectively. In these two models, isothermality (*b* = 0.0120–0.0123) and soil organic carbon content at 15–30 cm (*b* = 0.0107–0.0122) were significant predictors.

Correlation analysis between these environmental drivers and the genus-level co-occurrence network revealed specific associations (Figure 6B,C). Soil organic carbon (soc_15.30cm_mean.all) was significantly negatively correlated with the relative abundances of *Actinomyces*, *Muribaculum*, *unclassified_f__Eggerthellaceae*, *Clostridioides*, *Vibrio*, *Eggerthella*, *Corynebacterium*, *Phocaeicola*, *Lactobacillus*, and *Zhenpiania* (*p* < 0.05). Meanwhile, bio_03 was significantly positively correlated with the relative abundances of *Actinomyces*, *Muribaculum*, *unclassified_f__Eggerthellaceae*, *Clostridioides*, and *Vibrio* (*p* < 0.05). Taken together, these factors may influence the gut microbial community structure of goitered gazelle by affecting key functional nodes within the network.

### 3.6. Environmental Drivers of Gut Microbial Function Variations

**Figure 7:**
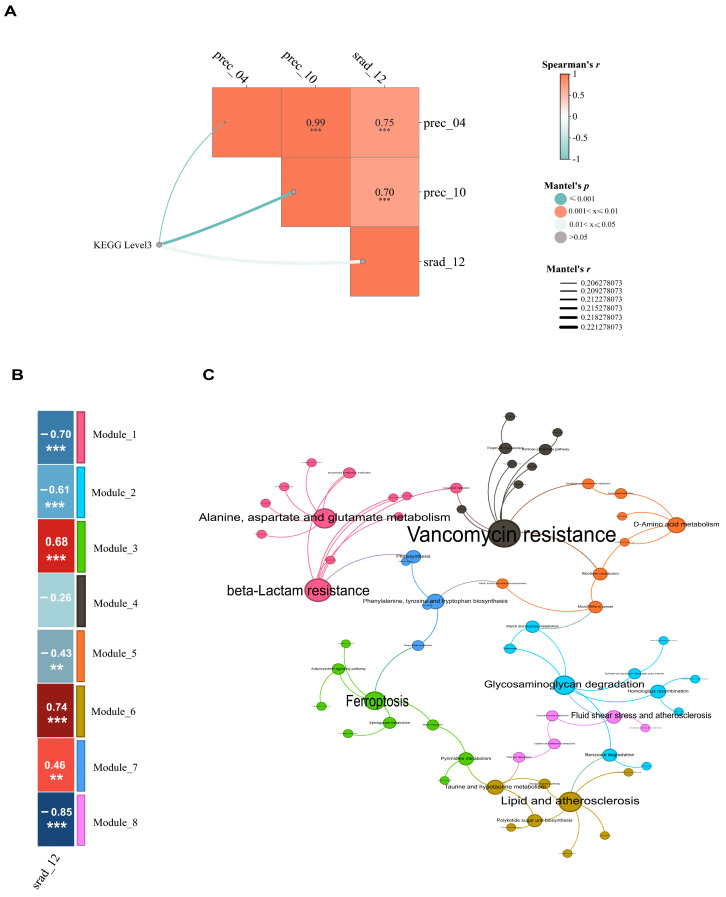
(**A**) Environmental factors significantly correlated with variation in KEGG level 3 functional profiles in gut microbiome of goitered gazelles. (**B**) Spearman correlation heatmap between the eigengenes of the identified network modules and srad_12, gut microbial composition, where ** *p* < 0.01, *** *p* < 0.001. (**C**) Co-occurrence network of KEGG level 3 functional profiles in goitered gazelles. Node colors indicate the module to which each node belongs, and node size and label font size represent the node’s PageRank value, reflecting its overall importance within the network.

Mantel tests were performed to examine the correlations between environmental factors and the Bray–Curtis distances derived from KEGG level 3 functional profiles of the goitered gazelle gut microbiota (Figure 7A). The analysis identified three environmental factors with significant correlations (Mantel test, *p* < 0.05): solar radiation-related factors (srad_12) and precipitation-related factors (prec_04, prec_10). Subsequently, two factors—srad_12, and the gut microbial composition—were selected for inclusion in the predictive modeling.

To predict variations in KEGG functional profiles of the gut microbiota among different goitered gazelle populations, a multiple regression on matrices (MRM) predictive model was constructed. The results of multivariable modeling indicated that all fitted models were significant (*p* < 0.05), with Model 1 explaining 28.6% of the variance (Table 2). The analysis demonstrated that both srad_12 and the gut microbial community structure were significant predictors of the functional similarity in KEGG level 3 functional profiles across the different populations.

At KEGG level 3, pathways significantly negatively correlated with srad_12 included beta-Lactam resistance; Alanine, aspartate and glutamate metabolism; and Glycosaminoglycan degradation (*p* < 0.05), pathways showing significant positive correlations with srad_12 were Ferroptosis; Phenylalanine, tyrosine and tryptophan biosynthesis; and Lipid and atherosclerosis (*p* < 0.05) (Figure 7B,C).

## 4. Discussion

### 4.1. The Core Gut Microbiome: Implications for Gazelle Nutrient Acquisition in the Qaidam Basin

This study identified *Firmicutes* and *Bacteroidota* as the dominant bacterial phyla in the gut microbiota of goitered gazelles, collectively constituting over 75% of the relative abundance. *Firmicutes* are known for cellulose degradation, while *Bacteroidota* contribute to the breakdown of starch, pectin, and xylan [42]. A similar phylum-level composition has been reported in other plateau herbivores, including the Tibetan wild ass (*Equus kiang*) [43], Yak (*Bos grunniens*) [44], and Tibetan sheep (*Ovis aries*) [45], underscoring the conserved and crucial role these phyla play in maintaining gut homeostasis and host health in ungulates inhabiting high-altitude regions [45]. At the genus level, relatively high abundances of *Ruminococcus*, *Bacteroides*, *Alistipes*, and *Eubacterium* were observed. These genera are pivotal for plant-fiber degradation and short-chain fatty acid (SCFA) production [46], processes essential for energy acquisition in ruminants. Their prevalence suggests a microbial community structure that facilitates metabolic efficiency, potentially supporting the goitered gazelles to thrive on the low-quality, high-fiber forage typical of arid plateau ecosystems [47].

Functionally, metabolic pathways, particularly those for carbohydrate and amino acid metabolism, were highly enriched within the gut microbiota. This functional profile is consistent with the breakdown of complex plant fibers and nutrient absorption [48], which is directly congruent with the wild gazelles’ diet, predominantly composed of high-fiber grasses from families such as *Poaceae* and *Chenopodiaceae* [49]. Overall, these core taxonomic and functional features underscore the microbiome’s role in meeting the host’s nutritional demands within an extreme environment.

### 4.2. Isothermality and Soil Organic Carbon as Key Drivers of Microbial Variation

Predictive modeling identified isothermality (bio_03) and soil organic carbon content at 15–30 cm depth (soc_15.30cm_mean.all) as the most significant predictors of gut microbial community variation in goitered gazelles, explaining 19% of the microbial variation. While this explanatory power is relatively modest, it is consistent with many wildlife studies where a substantial portion of microbial variance is typically attributed to unmeasured factors, such as host genetics, individual health, and stochastic assembly processes. Nonetheless, these results highlight the critical environmental axes associated with gut microbial shifts in the Qaidam Basin.

Isothermality reflects the stability of diurnal and seasonal temperature fluctuations [50]. Higher values indicate a more stable thermal environment, which favor stable plant growth cycles and the accumulation of nutrients. Isothermality (bio_03) is a key climatic determinant for the distribution of goitered gazelles [51]. Such stability reduces the host’s energetic costs for thermoregulation and is often associated with enhanced vegetation diversity and more predictable plant growth cycles [52], thereby optimizing diet quality. Given that diet is a primary driver of gut microbiota composition—often surpassing host genetics [53,54], especially in herbivores—isothermality likely is associated with the gut microbiome indirectly by modulating the quantity, quality, and diversity of available forage. 

Soil organic carbon may be linked to the gut microbiota through multiple interconnected pathways. Firstly, it directly fuels soil microbial activity [55,56], and improves soil physicochemical properties, thereby promoting plant biomass production and nutrient allocation [57,58]. Secondly, and perhaps more critically, soil microbial communities constitute a vital environmental “seed bank” for plant-associated microbiomes [59,60,61]. During grazing, microbes from plant surfaces and tissues are ingested, serving as an exogenous inoculum for the gazelle’s gut. This link is particularly strong in goitered gazelles, which acquire a notably high proportion (1.8–8.9%) of their gut microbes from environmental sources compared to other species on the Qinghai–Tibetan Plateau [62]. Thus, the statistical association between soil organic carbon and gut microbiota likely reflects this cascading influence from soil to plant to herbivore.

The observed regional variations in gut microbial alpha-diversity further align with these indirect mechanisms. The MA region, characterized by sparse vegetation, low precipitation, and poor soils [49,63], exhibited the lowest microbial diversity. This suggests that limited forage availability may lead to both substrate scarcity and reduced exogenous microbial input. Additionally, reliance on soil-derived microbes in such harsh environments may increase exposure to soil contaminants (e.g., antibiotic residues) [62], potentially suppressing sensitive microbial taxa and reduce α-diversity [64]. Conversely, the KLK region, with more favorable climatic and soil conditions that support richer vegetation [49,63], hosted the highest gut microbial diversity. This aligns with the premise that abundant and diverse plant resources provide a richer substrate base and a more stable environment for microbial colonization, sustaining higher diversity and functional potential.

### 4.3. Gut Microbial Composition and Winter Solar Radiation Co-Drive Functional Variation

Predictive modeling of gut microbial functional variation identified two key drivers: gut microbial composition and December solar radiation (srad_12).

Changes in the *Firmicutes*-to-*Bacteroidota* ratio are often associated with shifts in host metabolic functions [65]. Our finding of a strong compositional influence on function aligns with the “structure–function co-variation” concept [66]. This contrasts with the study by Qin et al., which reported highly similar functional profiles between goitered gazelles and domestic sheep despite distinct microbial compositions [67]. Such discrepancies may arise from differences in study scale, host ecological niches, or the specific environmental pressures examined.

A notable finding was the significant association between December solar radiation and microbial functional potential, despite its lack of a direct effect on community composition. We posit that in the high-altitude, arid Qaidam Basin, winter solar radiation—a key environmental factor [68] —exerts an indirect influence. It likely modulates vegetation composition, primary productivity, and the synthesis of plant secondary metabolites [69], thereby altering the dietary substrate quality available to the host and, consequently, the metabolic demands on the gut microbiota [70].

At the KEGG level 3, network analysis revealed that gut microbial functional modules exhibited divergent association patterns with environmental variables. Pathways involved in core amino acid turnover and complex carbohydrate degradation (e.g., Alanine, aspartate and glutamate metabolism) showed negative correlations with solar radiation, while modules representing stress-responsive regulatory processes (e.g., Phenylalanine, tyrosine and tryptophan biosynthesis) demonstrated positive correlations. These patterns suggest an environment-associated redistribution of microbial metabolic priorities. Therefore, we infer that during winter, the gut microbiota of goitered gazelles may undergo a functional transition toward increased stress-response activity while fine-tuning core metabolism. This shift is potentially linked to optimized energy utilization from a limited diet and may support immune capacity [71], representing a potential functional response to seasonal resource scarcity that assists the host in maintaining metabolic homeostasis.

## 5. Conclusions

This study indicates that in the extreme environment of the Qaidam Basin, the gut microbiota of the goitered gazelle is associated with two essential roles: maintaining a core functional stability for efficient nutrient extraction and displaying functional plasticity in response to specific environmental pressures. This dual capacity—to preserve metabolic homeostasis while dynamically tuning community structure and metabolism—suggests potential relevance in supporting host ecological responses to environmental pressures. Consequently, our findings reframe our understanding of environment-associated variation in this keystone species: from a primarily host-centric trait to a dynamic homeostasis sustained by the interplay between host, microbiome, and environment. This perspective is pivotal for forecasting ecological resilience and developing conservation strategies that incorporate critical microbial feedbacks in the face of ecosystem change.

## Figures and Tables

**Table 1:** Multivariate MRM analysis of variation in genus-level gut microbial composition of goitered gazelles.

Module No.	Model R^2^ Value	Model *p* Values	Predictor	coef (*b*)	*p*-Values
Mod1	0.191	0.0001	bio_03	0.0123	0.0001
			soc_15.30cm_mean.all	0.0122	0.0010
			nitrogen_15.30cm_mean.all	−0.002	0.139
Mod2	0.190	0.0001	bio_03	0.0120	0.0001
			soc_15.30cm_mean.all	0.0107	0.0007

**Table 2:** Multivariate MRM analysis of variation in KEGG level 3 functional profiles of goitered gazelle gut microbiota.

Module No.	Model R^2^ Value	Model *p* Values	Predictor	coef (*b*)	*p*-Values
Mod1	0.286	0.0001	srad_12	0.0034	0.0311
			gut microbial composition	0.2355	0.0001

## Data Availability

The raw metagenomic sequencing data generated in this study have been deposited in the Genome Sequence Archive at the National Genomics Data Center, China National Center for Bioinformation/Beijing Institute of Genomics, Chinese Academy of Sciences (https://ngdc.cncb.ac.cn/bioproject/, bioproject: PRJCA049756, accessed on 1 December 2025).
